# Regarding Article 'Multiple Episodes of Presyncope in a Pacemaker Dependent Patient: What is the Diagnosis?'

**Published:** 2010-07-20

**Authors:** Kartikeya Bhargava

**Affiliations:** Division of Electrophysiology, Medanta Heart Institute, Medanta, The Medicity

**Keywords:** Presyncope, Pacemaker

I read with interest the article describing repetitive non-reentrant ventriculo-atrial synchrony (RNRVAS) as an explanation for the recorded marker channels and presyncope in a pacemaker dependent patient [1]. Though, the mechanism explained is plausible, I suggest an alternative and more common mechanism as a likely explanation of the intra-cardiac recording.

Before dwelling with the mechanism itself, lets look at some of the facts that appear from the recording after precise measurements:

1. The paced AV delay (PAV) depicted in the recording varies from 109 to 132 ms though the programmed PAV was 200 ms. Rate-adaptive AV delay (if programmed on) may explain this shortening, though marked shortening and marked variability can be explained only by non-competitive atrial pacing (NCAP) algorithm.
2. The interval that from AR to AP is fixed at 296 ms and indicates operation of NCAP algorithm.

NCAP algorithm is designed to prevent competitive atrial pacing in Medtronic dual-chamber pacemakers and is nominally 'on'. It is activated on sensing atrial activity (AR) in the post-ventricular atrial refractory period. AR event does not trigger a ventricular pace. Also, NCAP algorithm delays the A pace for 300 ms (non-programmable in pacemakers) after AR even though, lower rate or sensor indicated rate may have required atrial pace to occur earlier. This prevents atrial pacing when the atrial tissue is refractory and thus may reduce atrial arrhythmias. The interval 300 ms is chosen based on the observations that an atrial stimulus delivered earlier than 300 ms may find the atrial myocardium refractory. But the present case and another report [2] suggest that the atrial myocardium especially if diseased may not recover excitability in this period. In the NCAP algorithm, the pacemaker also attempts to maintain the ventricular pacing rate at the sensor indicated rate (or the lower rate). This is done by shortening the PAV as is seen in the present example.

Now, for a diagnosis of RNRVAS, it has to be shown that the AR events were due to retrograde VA conduction. This can be verified if one finds that the atrial EGM represented by AR has different morphology than that of sinus events. 

RNRVAS requires intact VA conduction, which though possible, is unusual in a patient with antegrade permanent complete AV block. In the present case, though RNRVAS is plausible, it is also possible that all the AR events were sinus events and NCAP algorithm with a higher sensor indicated rate resulted in the recorded high atrial rate event. This can be initiated by a late atrial premature event falling in PVARP or a ventricular premature event leading to next sinus beat falling in PVARP during DDDR pacing at a high sensor indicated rate (higher than sinus rate) and operation of NCAP algorithm. The first AR event will lead all subsequent sinus events to fall in the PVARP and a similar recording.

Whatever, may be the mechanism of the recording, turning 'off' NCAP algorithm and reducing the PVARP are likely to help in preventing the occurrence of similar episode in future.
